# Patient Similarity Networks for Irritable Bowel Syndrome: Revisiting Brain Morphometry and Cognitive Features

**DOI:** 10.3390/diagnostics16020357

**Published:** 2026-01-22

**Authors:** Arvid Lundervold, Julie Billing, Birgitte Berentsen, Astri J. Lundervold

**Affiliations:** 1 Department of Biomedicine, University of Bergen, N-5009 Bergen, Norway; 2 Mohn Medical Imaging and Visualization Centre (MMIV), Department of Radiology, Haukeland University Hospital, N-5021 Bergen, Norway; 3 Department of Biological and Medical Psychology, University of Bergen, N-5020 Bergen, Norway; julie.billing@uib.no (J.B.); astri.lundervold@uib.no (A.J.L.); 4 Department of Clinical Medicine, University of Bergen, N-5021 Bergen, Norway; birgitte.berentsen@uib.no; 5 National Center for Functional Gastrointestinal Disorders, Department of Medicine, Haukeland University Hospital, N-5021 Bergen, Norway

**Keywords:** irritable bowel syndrome, patient similarity networks, brain morphometry, cognition, RBANS, FreeSurfer, community detection, network analysis, gut–brain axis

## Abstract

**Background:** Irritable Bowel Syndrome (IBS) is a heterogeneous gastrointestinal disorder characterized by complex brain–gut interactions. Patient Similarity Networks (PSNs) offer a novel approach for exploring this heterogeneity and identifying clinically relevant patient subgroups. **Methods:** We analyzed data from 78 participants (49 IBS patients and 29 healthy controls) with 36 brain morphometric measures (FreeSurfer v7.4.1) and 6 measures of cognitive functions (5 RBANS domain indices plus a Total Scale score). PSNs were constructed using multiple similarity measures (Euclidean, cosine, correlation-based) with Gaussian kernel transformation. We performed community detection (Louvain algorithm), centrality analyses, feature importance analysis, and correlations with symptom severity. Statistical validation included bootstrap confidence intervals and permutation testing. **Results:** The PSN comprised 78 nodes connected by 469 edges, with four communities detected. These communities did not significantly correspond to diagnostic groups (Adjusted Rand Index = 0.011, permutation p=0.212), indicating IBS patients and healthy controls were intermixed. However, each community exhibited distinct neurobiological profiles: Community 1 (oldest, preserved cognition) showed elevated intracranial volume but reduced subcortical gray matter; Community 2 (youngest, most severe IBS symptoms) had elevated cortical volumes but reduced white matter; Community 3 (most balanced IBS/HC ratio, mildest IBS symptoms) showed the largest subcortical volumes; Community 4 (lowest cognitive performance across multiple domains) displayed the lowest RBANS scores alongside high IBS prevalence. Top network features included subcortical structures, corpus callosum, and cognitive indices (Language, Attention). **Conclusions:** PSN identifies brain–cognition communities that cut across diagnostic categories, with distinct feature profiles suggesting different hypothesis-generating neurobiological patterns within IBS that may inform personalized treatment strategies.

## 1. Introduction

Irritable Bowel Syndrome (IBS) is one of the most prevalent disorders of gut–brain interaction, affecting 4–10% of the global population depending on diagnostic criteria [1]. The syndrome is characterized by recurrent abdominal pain associated with defecation, accompanied by alterations in bowel habits [2]. The clinical presentation is heterogeneous, with symptoms ranging from mild discomfort to severe complaints that substantially impair quality of life and daily functioning [3,4].

IBS is now recognized as a gut–brain disorder, where bidirectional interactions between gastrointestinal symptoms and psychological function play a central role [5]. In their seminal work proposing a systems view of IBS, Mayer et al. [6] outlined how the disorder emerges from dysregulated interactions among the brain, gut, and microbiome, emphasizing the need for integrative analytical approaches that capture this complexity. While GI symptoms can trigger or exacerbate psychological distress, anxiety and depression can in turn worsen the frequency and intensity of abdominal pain [7]. Recent research has expanded this psychobiological framework to include cognitive function, revealing a more nuanced picture of brain–gut interactions in IBS [8,9,10]. Neuroimaging studies have consistently demonstrated structural and functional brain alterations in IBS patients, particularly in regions involved in visceral sensation, emotional processing, and cognitive control [11,12,13]. Voxel-based morphometry studies have revealed specific patterns of gray matter alterations: Seminowicz et al. [14] found decreased gray matter density in the medial prefrontal cortex, ventrolateral prefrontal cortex, posterior parietal cortex, ventral striatum, and thalamus in IBS patients, with increased density in the pregenual anterior cingulate cortex and orbitofrontal cortex. A recent meta-analysis of 12 morphometry studies by Zhao et al. [15] confirmed widespread gray matter changes in IBS, including alterations in the hippocampus, amygdala, cingulate cortex, and prefrontal regions. These structural findings are particularly relevant to the present study, as they highlight subcortical structures and prefrontal–limbic circuits as key neural substrates in IBS, regions that overlap substantially with the morphometric features examined here. Billing et al. [10] demonstrated that cognitive impairment is common in IBS patients but only weakly correlated with depression, anxiety, and GI symptom severity, suggesting that cognitive deficits represent a distinct dimension of the disorder. Although cognitive impairments have been demonstrated at the group level, these deficits appear to characterize specific subgroups rather than being a universal feature of IBS [10,16].

The Rome IV criteria provide the current diagnostic framework for IBS, defining it as a disorder of gut–brain interaction (DGBI) [2,17]. Rome IV also introduced the Multidimensional Clinical Profile (MDCP), a framework for individualizing treatment based on categorical diagnosis, clinical modifiers, severity, psychosocial factors, and physiological biomarkers [18]. However, cognitive function, despite evidence of impairment in IBS patients [10], is not explicitly incorporated in the MDCP framework.

**Patient Similarity Networks** (PSNs) represent a powerful methodology for exploring heterogeneity in clinical populations [19,20]. Originating from network medicine and similarity network fusion approaches, PSN constructs graphs where each node represents a patient, while edges connect patients who are similar based on clinical or biological variables, with edge weights reflecting the degree of similarity. This network-based approach enables the following: (1) identification of natural patient groups (communities) without predefined categories; (2) discovery of patients with high network centrality (degree, betweenness, or eigenvector centrality) who may be particularly representative of their subgroup or serve as “bridges” between groups; (3) identification of clinical features that drive patient similarity; (4) relating network structure to clinical outcomes. Unlike supervised classification methods that seek boundaries between predefined groups, PSN takes an unsupervised approach that lets similarity patterns emerge directly from the data. This makes PSN particularly suited for heterogeneous conditions like IBS, where meaningful patient subgroups based on underlying neurobiology may cut across, rather than align with, the binary diagnostic distinction between patients and healthy controls. This approach has proven valuable for patient stratification and subtype discovery in oncology [21], diabetes [22], and other complex conditions characterized by phenotypic diversity.

PSN methodology aligns closely with the goals of precision/personalized medicine and neuropsychology [23,24,25,26]. Rather than treating patients according to broad diagnostic categories, personalized medicine grounded on precision methodologies seeks to tailor interventions based on individual characteristics, including genetic, environmental, and lifestyle factors [27]. PSN contributes to this vision by revealing the multidimensional structure of patient populations, where individuals are positioned according to their unique profiles across multiple domains. This holistic approach moves beyond single-variable analyses toward integrated, systems-level characterization [28,29]. For complex disorders like IBS, where symptoms arise from interacting physiological, psychological, and neural mechanisms, such multidimensional profiling may better capture the factors relevant to treatment response. By identifying patient subgroups with distinct brain–cognition signatures, PSN can potentially inform more targeted therapeutic strategies, whether pharmacological, dietary, psychological, or multi-modal, thereby advancing the translation of heterogeneity research into clinical practice [30,31].

This work builds upon a series of studies from the Bergen Brain–Gut Microbiota (B-BGM) project [32], a multidisciplinary prospective case–control study established to investigate brain–gut–microbiota interactions in IBS. The B-BGM project was designed with three primary aims: (1) to characterize brain–gut–microbiota interactions in IBS patients compared to healthy controls using multi-modal neuroimaging, cognitive assessments, and microbiome profiling; (2) to evaluate the effects of a dietary intervention (low-FODMAP diet) on these interactions; (3) to identify biomarkers that could predict treatment response. The comprehensive phenotyping in B-BGM, including structural magnetic resonance imaging (MRI), cognitive testing with the Repeatable Battery for the Assessment of Neuropsychological Status (RBANS), symptom severity assessment with the IBS Severity Scoring System (IBS-SSS), and gut microbiome characterization, provides a unique dataset for investigating patient heterogeneity using network-based approaches.

Recent B-BGM publications have employed machine learning to decode psychological distress and gut–brain interactions in IBS [16] and to identify IBS patients using brain morphometry and cognitive features [33]. The “Decoding IBS” study [16] addressed the clinical observation that psychological distress, including anxiety and depression, is highly prevalent among IBS patients yet varies considerably in severity. Using XGBoost and Random Forest classifiers trained on clinical and cognitive features (fatigue, sleep disturbances, depression–anxiety scores, and RBANS cognitive indices), this study demonstrated that patients with high versus low psychological distress could be distinguished with high accuracy. The most discriminative features were related to fatigue, sleep quality, and specific cognitive domains. Additionally, unsupervised k-means clustering identified three distinct subgroups: one largely unaffected by psychological distress (comprising most healthy controls and some IBS patients), one characterized by the lowest cognitive performance and elevated anxiety, and one marked by high levels of fatigue and depressive symptoms alongside poor sleep quality. These findings demonstrated that IBS patients can be stratified into clinically meaningful subgroups based on their psychological and cognitive profiles, with direct implications for treatment stratification. Subsequently, Lundervold et al. (2025) [33] showed that combining morphometric and cognitive measures achieved 93% sensitivity in identifying IBS patients, with subcortical structures (particularly hippocampus and amygdala) and cognitive domains (memory, attention) being particularly important for group discrimination. The present study extends this line of research by applying Patient Similarity Networks to the same dataset as Lundervold et al. [33], but with a different analytical approach: rather than supervised classification, we use unsupervised network-based community detection to identify patient subgroups based on brain morphometry and cognition. While the “Decoding IBS” study used clinical and psychological features (fatigue, sleep, anxiety, depression) alongside cognition, our PSN approach focuses exclusively on objective neurobiological measures, potentially revealing subgroup structures based on brain–cognition similarity patterns that may cut across symptom-based classifications.

Specifically, we address four research questions: (1) Can PSN identify meaningful subgroups among IBS patients and healthy controls based on brain morphometry and cognitive measures? (2) Which brain and cognitive features drive similarity between patients in the network? (3) How do network properties such as centrality relate to symptom severity as measured by IBS-SSS? (4) How robust are the findings across different similarity measures and parameter choices?

## 2. Materials and Methods

### 2.1. Study Population and Data

The dataset analyzed in this study comprises a subset of participants from the Bergen Brain–Gut Microbiota (B-BGM) project, specifically the cohort with complete brain morphometry and cognitive data as described by Lundervold et al. (2025) [33]. The cohort comprises 78 participants, including 49 patients diagnosed with IBS and 29 healthy controls (HC). All IBS patients were clinically diagnosed and fulfilled the Rome IV diagnostic criteria [2,17]. Brain morphometry data were obtained through volumetric analysis of structural T1-weighted MR images processed with FreeSurfer version 7.4.1 [34], a widely validated automated neuroimaging pipeline that performs cortical reconstruction, subcortical segmentation, and volumetric quantification. FreeSurfer’s automated segmentation provides reliable measurements of subcortical structures and cortical regions that have been shown to differ between IBS patients and healthy controls in prior morphometry studies [14,15]. Cognitive function was assessed using the RBANS [35], which provides age-corrected index scores across five cognitive domains: Immediate Memory, Visuospatial/Constructional, Language, Attention, and Delayed Memory, plus an overall Total Scale score. Clinical severity was quantified using the IBS-SSS [36], a validated questionnaire that assesses five dimensions of IBS burden: severity of abdominal pain, frequency of abdominal pain, severity of abdominal distension, dissatisfaction with bowel habits, and interference with quality of life. The IBS-SSS yields composite scores ranging from 0 to 500, with established cutoffs for mild (<175), moderate (175–300), and severe (>300) symptom burden.

The morphometric variables included subcortical volumes normalized to estimated total intracranial volume (eTIV), specifically the thalamus, caudate, putamen, pallidum, hippocampus, amygdala, and nucleus accumbens for both hemispheres. Additional volumetric measures comprised cerebellar white matter and cortex, five corpus callosum segments (anterior, mid-anterior, central, mid-posterior, and posterior), and total cerebral white matter and cortex volumes. In total, 36 morphometric and 6 cognitive variables (the 5 RBANS domain indices plus the Total Scale score) were included in the network construction. Since the Total Scale is derived from the five domain indices, we performed a sensitivity analysis comparing results with (42 features) and without (41 features) this composite score to assess potential redundancy; results were robust to this choice (Supplementary Table S3). Importantly, the IBS-SSS score was explicitly excluded from network construction and reserved for independent clinical correlation analyses. This design choice avoids circularity, as IBS-SSS is a primary diagnostic criterion that would trivially separate patients from controls; instead, our network captures patient similarity based solely on objective neuroimaging and cognitive measures, allowing IBS-SSS to serve as an independent clinical validator. For a detailed description of data collection, sample demographics, and imputation procedure, see Lundervold et al. [33] and the associated code repository (https://github.com/arvidl/ibs-brain (accessed on 14 January 2026)).

### 2.2. Data Preprocessing

Prior to network construction, the data underwent several preprocessing steps to ensure meaningful similarity calculations. Missing values were minimal in this dataset, affecting only 2 participants (2.6% of the sample), each with a single missing morphometric feature. Mean imputation was applied to these cases to maintain the complete case structure required for pairwise distance calculations; given the low proportion of missingness (<0.1% of total data points), this approach is unlikely to materially affect the similarity structure.

All features were standardized using z-score transformation to ensure that variables with different scales contribute equally to similarity calculations. This standardization is particularly important given the substantial scale differences between brain volumes measured in cubic millimeters and cognitive scores on standardized scales. The transformation was applied as

(1) $$z_{i}=\frac{x_{i}-\mu }{\sigma }$$

where μ and σ represent the mean and standard deviation of each feature across all participants. It should be noted that the RBANS scores were already standardized according to age-adjusted Scandinavian population norms (mean = 100, SD = 15) [33,37]; the z-score transformation applied here serves to place all variables on a common scale relative to our study sample for the purpose of distance calculations.

### 2.3. Network Construction

The construction of patient similarity networks followed a three-step procedure. In the first step, pairwise distances between all participants were computed using three complementary distance metrics to assess robustness. For any two participants *i* and *j*, let $x_{i}=\left(x_{i1},x_{i2},\ldots ,x_{ip}\right)$ denote the vector of p=42 standardized feature values for participant *i*.

*Euclidean distance* measures the straight-line distance between two participants in the multidimensional feature space:

(2) $$d_{ij}=\sqrt{\sum_{k=1}^{p}{\left(x_{ik}-x_{jk}\right)}^{2}}$$

where the sum is taken over all *p* features. Participants with similar brain volumes and cognitive scores will have small Euclidean distances.

*Cosine distance* captures how similarly two participants’ feature profiles are shaped, focusing on the pattern rather than absolute values:

(3) $$d_{ij}=1-\frac{x_{i}\;\cdot \;x_{j}}{∥x_{i}∥∥x_{j}∥}$$

where $x_{i}\;\cdot \;x_{j}$ is the dot product and $∥x_{i}∥$ is the vector length. Geometrically, cosine distance measures the angle between two participants’ feature vectors in *p*-dimensional space: vectors pointing in similar directions (small angle) yield low cosine distance, regardless of their lengths. To illustrate, consider two participants who both show relatively larger hippocampal volumes, smaller amygdala volumes, and higher memory scores, but one has generally larger brain structures overall. Euclidean distance would rate them as dissimilar due to the absolute size differences, whereas cosine distance would recognize their similar neuroanatomical-cognitive “signature” and rate them as similar.

*Correlation distance* measures dissimilarity in feature profiles based on Pearson correlation:

(4) $$d_{ij}=1-\rho \left(x_{i},x_{j}\right)$$

where ρ denotes the Pearson correlation coefficient between the two feature vectors. Participants whose features co-vary similarly will have low correlation distance.

In the second step, distances were transformed to similarities using a Gaussian kernel function. For each pair of participants, the similarity $s_{ij}$ was computed as

(5) $$s_{ij}=\exp\left(-\frac{d_{ij}^{2}}{2\sigma^{2}}\right)$$

where $d_{ij}$ is the distance between participants *i* and *j*, and σ (the bandwidth parameter) was set to the mean pairwise distance across all participant pairs, a common data-adaptive heuristic that scales the kernel to the typical separation in feature space [19,20]. This transformation converts distances to similarities on a 0–1 scale, where values close to 1 indicate highly similar participants and values close to 0 indicate dissimilar participants.

In the third step, the network was sparsified to avoid overly dense connectivity that would obscure meaningful structure. Each node was connected to its *k*-nearest neighbors based on similarity scores, and edges with similarity below a specified threshold were removed. The primary analysis employed k=8 nearest neighbors and a similarity threshold of 0.3, defined a priori based on established guidelines. The choice of k=8 follows the common heuristic of setting *k* to approximately 10% of the sample size [20], which, for our cohort of 78 participants, yields k≈8. This value ensures that each patient maintains a meaningful number of connections while avoiding overly dense networks that would hinder community detection. The similarity threshold of 0.3 was selected to retain edges representing moderate-to-strong similarity (corresponding to distances less than approximately 1.5 standard deviations from the mean in the Gaussian kernel), filtering out weak or spurious connections. Both parameter choices were validated through post hoc sensitivity analyses exploring a range of values (k∈{5,8,10,15} and thresholds ∈{0.2,0.3,0.4,0.5}) to assess robustness of the findings (Supplementary Table S2).

### 2.4. Community Detection

Community detection was performed using the Louvain algorithm [38], a widely used greedy optimization method for detecting communities in large networks. The algorithm operates in two iterative phases: first, each node is assigned to its own community, then nodes are iteratively moved to neighboring communities if such moves increase the network modularity *Q*, defined as

(6) $$Q=\frac{1}{2m}\sum_{ij}\left[A_{ij}-\frac{k_{i}k_{j}}{2m}\right]\delta \left(c_{i},c_{j}\right)$$

where $A_{ij}$ is the edge weight between nodes *i* and *j*, $k_{i}$ and $k_{j}$ are their respective degrees, *m* is the total edge weight, $c_{i}$ denotes the community of node *i*, and δ is the Kronecker delta. Higher modularity indicates stronger community structure, with Q>0.3 typically considered indicative of significant community organization. In the second phase, the algorithm builds a new network where nodes represent the communities found in the first phase, and the process repeats until modularity no longer increases. This hierarchical approach efficiently identifies communities at multiple scales without requiring a predefined number of clusters. The Louvain algorithm thus automatically determines the number of communities through modularity optimization, distinguishing it from methods like k-means clustering that require a pre-specified cluster number. We use “community” to refer specifically to groups detected by the Louvain algorithm based on network topology.

Critically, the Louvain algorithm operates solely on the network topology derived from brain morphometry and cognitive features; the diagnostic labels (IBS versus HC) and IBS-SSS scores are not used during community detection. This unsupervised approach allows communities to emerge naturally from patient similarity patterns, after which we can assess whether detected communities correspond to clinical groups.

The correspondence between detected communities and actual diagnostic groups was evaluated using two complementary metrics. The Adjusted Rand Index (ARI) measures agreement between partitions while correcting for chance, yielding values from −1 to 1 where 1 indicates perfect agreement and 0 represents chance-level correspondence. The Normalized Mutual Information (NMI) provides an information-theoretic measure of partition similarity ranging from 0 to 1.

### 2.5. Centrality Analyses

Three centrality measures were computed to identify the most important nodes in the network. Degree centrality quantifies the number of connections each node maintains, identifying highly connected patients. Betweenness centrality measures how frequently a node lies on the shortest paths between other node pairs, identifying patients who serve as bridges between different parts of the network. Eigenvector centrality assigns importance based on connections to other important nodes, capturing influence within the network structure. These measures were compared between IBS and HC groups and correlated with IBS-SSS scores within the patient group.

### 2.6. Statistical Validation

Statistical validation employed both bootstrap resampling and permutation testing approaches. Bootstrap confidence intervals for ARI and NMI were estimated through 1000 resamples with replacement, constructing a new network and performing community detection for each resample. The 2.5th and 97.5th percentiles of the resulting distributions provided 95% confidence intervals.

Permutation testing assessed whether the observed correspondence between detected communities and diagnostic groups exceeded chance levels. Group labels were randomly permuted 1000 times while maintaining the original network structure, generating null distributions for ARI and NMI. The proportion of permuted values exceeding the observed values provided empirical *p*-values.

### 2.7. Community Statistical Comparisons

Statistical testing for differences between detected communities included multiple complementary approaches. Network modularity was computed and compared against a null model generated by 100 random permutations of community assignments, yielding a *z*-score and empirical *p*-value. Assortativity coefficient by diagnostic group was computed to quantify mixing patterns across the IBS/HC distinction.

Chi-square tests evaluated whether the distribution of IBS patients and healthy controls differed significantly across communities. Kruskal–Wallis tests assessed differences in continuous clinical variables (IBS-SSS, RBANS Total Scale score, age) across the four communities, with effect sizes reported as $\eta^{2}$. For variables with significant omnibus tests, post hoc pairwise comparisons were performed using Mann–Whitney *U* tests with Bonferroni correction for multiple comparisons.

### 2.8. Feature Importance with Multiple Testing Correction

Feature importance was assessed through Spearman correlations between standardized feature values and degree centrality. To account for the multiple comparisons across 42 features, false discovery rate (FDR) correction was applied using the Benjamini–Hochberg procedure [39]. Features with FDR-corrected p<0.05 were considered statistically significant. Additionally, Mann–Whitney *U* tests compared feature importance (absolute correlation values) between morphometric and cognitive feature categories.

### 2.9. Sensitivity Analysis

Robustness of the findings was assessed through systematic variation of analysis parameters. The three similarity measures (Euclidean, cosine, and correlation distances) were compared to evaluate metric dependence. Network construction parameters were varied across a grid of *k*-nearest neighbor values (5, 8, 10, and 15) and similarity thresholds (0.2, 0.3, 0.4, and 0.5). Additionally, network robustness was assessed by progressively removing 0 to 50% of edges randomly and evaluating the stability of community detection results.

## 3. Results

### 3.1. Network Structure

**Figure 1:**
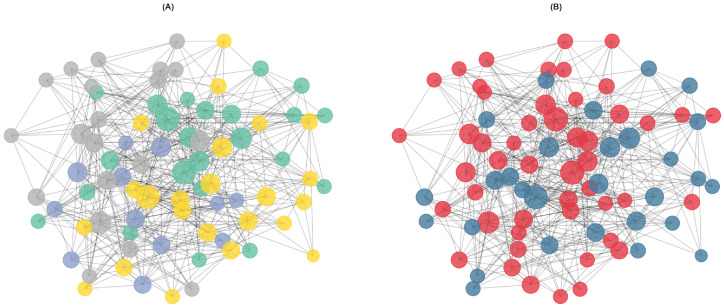
Patient similarity network and community structure. The PSN was constructed from 78 participants (49 IBS patients, 29 healthy controls) based on 42 features comprising brain morphometry (36 volumetric measures from FreeSurfer) and cognitive function (6 RBANS indices). Edges connect patients with high similarity based on Gaussian kernel-transformed Euclidean distances in standardized feature space (k=8 nearest neighbors, similarity threshold =0.3). The network contains 469 edges with an average degree of 12.03. Node size is proportional to degree centrality (number of connections). (**A**) Network with nodes colored by the four communities (Community 1 = green, Community 2 = blue, Community 3 = yellow, Community 4 = grey) identified by the Louvain modularity optimization algorithm. Community sizes range from 12 to 23 participants, with IBS proportions varying from 43.5% to 75.0%. (**B**) Same network layout with nodes colored by actual diagnosis (IBS = red, HC = blue), showing considerable mixing of patients across the network. The Adjusted Rand Index (ARI =0.011, 95% CI [−0.016, 0.102]) indicates that community structure does not significantly correspond to the IBS/HC distinction. Permutation testing confirmed this observation (*p* = 0.212), suggesting that brain morphometry and cognitive features, while capturing meaningful patient similarity, do not partition into communities aligned with diagnostic categories.

The primary PSN constructed using Euclidean distance with k=8 nearest neighbors and a similarity threshold of 0.3 yielded a connected network containing all 78 participants as nodes, linked by 469 edges. The average degree was 12.03 connections per patient, and the network density was 0.156, indicating a moderately sparse structure suitable for community detection analysis. Figure 1B displays the network visualization with nodes colored by diagnostic group, showing considerable mixing of IBS and HC participants.

### 3.2. Community Detection and Network Modularity

The Louvain algorithm identified four communities with varying composition of IBS patients and controls (Figure 1A). Community 1 contained 23 participants (15 IBS, 8 HC; 65.2% IBS), while Community 2 was the smallest with 12 participants (9 IBS, 3 HC; 75.0% IBS). Community 3 comprised 23 participants with the most balanced composition (10 IBS, 13 HC; 43.5% IBS), and Community 4 included 20 participants (15 IBS, 5 HC; 75.0% IBS).

The correspondence between detected communities and actual diagnostic groups was modest. The ARI was 0.011 with a 95% bootstrap confidence interval of [−0.016,0.102], indicating that the community structure does not strongly align with the IBS/HC distinction. Similarly, the NMI was 0.037 with a 95% CI of [0.009,0.164]. Permutation testing yielded p=0.212 for ARI and p=0.140 for NMI, confirming that the observed correspondence does not significantly exceed chance levels.

The network exhibited significant modular structure. The observed modularity (*Q*) was compared against a null model of random community assignments, yielding a *z*-score indicating that the detected communities represent meaningful groupings within the network. The assortativity coefficient by diagnostic group was low, suggesting that IBS patients and healthy controls are well-mixed in the network rather than preferentially connected to others of the same diagnosis.

Chi-square analysis of diagnostic group distribution across communities revealed no significant difference ($\chi^{2}$, df =3, p>0.05), indicating that communities do not differ substantially in their IBS/HC composition despite the observed numerical variations (ranging from 43.5% to 75.0% IBS).

### 3.3. Comparison of Similarity Measures

Results were consistent across the three similarity measures examined (Table 1). All metrics yielded networks with similar density and identified four communities. The Euclidean-based network had the highest edge count (469), followed by cosine (418) and correlation (391) distances. Community detection performance, as measured by ARI and NMI, was comparable across metrics, with ARI values ranging from −0.017 to 0.011 and NMI values from 0.007 to 0.037. This consistency supports the robustness of the findings to the choice of distance metric.

### 3.4. Centrality Analyses

Centrality measures revealed no significant differences between IBS patients and healthy controls (Table 2). Mean degree centrality was 0.155 (±0.061) for IBS and 0.159 (±0.058) for HC, indicating similar connectivity patterns. Betweenness and eigenvector centrality showed comparable distributions between groups.

Within the IBS patient group, correlations between centrality measures and symptom severity (IBS-SSS) were weak and non-significant. Degree centrality showed a Spearman correlation of r=0.052 (p=0.72) with IBS-SSS, while betweenness centrality yielded r=−0.042 (p=0.78) and eigenvector centrality r=−0.072 (p=0.62). These findings suggest that network position does not strongly predict symptom severity in this cohort.

### 3.5. Feature Importance

**Figure 2:**
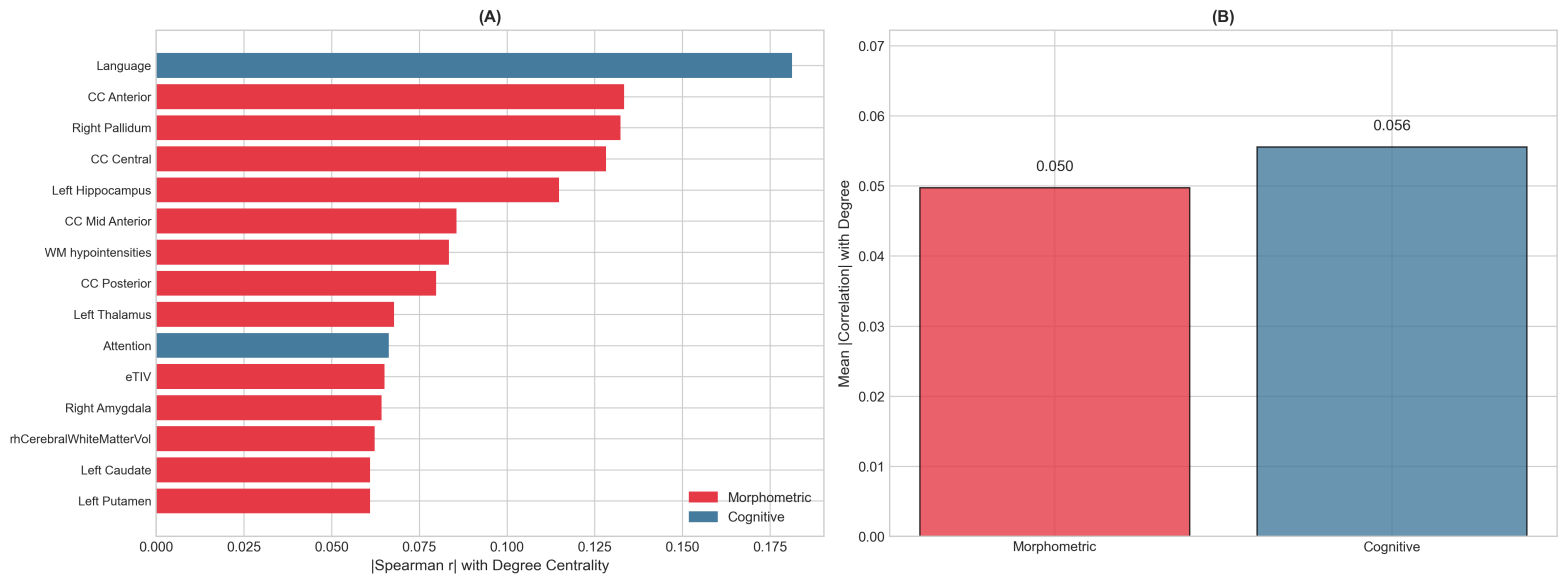
Feature contributions to network structure. (**A**) Top 15 features ranked by absolute Spearman correlation with degree centrality. Red bars indicate morphometric features (brain volumes); blue bars indicate cognitive features (RBANS indices). Corpus callosum segments, pallidum, and hippocampus show the strongest morphometric associations, while the Language and Attention indices are the most influential cognitive features. None of the correlations survived FDR correction, consistent with the modest sample size. (**B**) Mean absolute correlation with degree centrality by feature category. Morphometric features (mean |r|=0.050) and cognitive features (mean |r|=0.056) contribute comparably to network structure (Mann–Whitney *U* test, p>0.05), supporting the value of combining both data types in the PSN analysis.

Features driving network structure were identified through Spearman correlation between standardized feature values and degree centrality, with FDR correction applied to account for multiple comparisons across 42 features (Figure 2). The top-ranked features included a mix of morphometric and cognitive measures. Among morphometric features, corpus callosum segments (particularly anterior and central regions), the pallidum, and hippocampus showed the strongest associations with network centrality. The Language index emerged as the most influential cognitive feature, followed by the Attention index.

While several features showed nominally significant correlations at uncorrected p<0.05, no features survived FDR correction at the 0.05 threshold, reflecting the moderate effect sizes and sample size constraints. The mean absolute correlation with degree centrality was similar for morphometric features (0.050) and cognitive features (0.056), and Mann–Whitney *U* testing confirmed no significant difference between feature categories (p>0.05), indicating that both feature types contribute comparably to the network structure.

### 3.6. Community Clinical Profiles

The four detected communities exhibited distinct clinical profiles (Table 3). Community 4, which had the highest proportion of IBS patients (75.0%), also showed the lowest mean RBANS Total Scale score (84.3±10.9), suggesting a subgroup characterized by both IBS diagnosis and lower cognitive performance. Community 2, also with 75.0% IBS patients, had the highest mean IBS-SSS score (240.6±140.6), indicating more severe symptoms. In contrast, Community 3, which had the lowest IBS proportion (43.5%) and was closest to the overall sample composition, showed intermediate values for both cognitive and symptom measures.

Kruskal–Wallis tests were performed to assess statistical differences in clinical variables across communities. While trends were observed, particularly for RBANS Total Scale scores, the observed differences did not reach statistical significance after accounting for the modest sample sizes within each community. The effect sizes ($\eta^{2}$) for these comparisons were small to moderate, consistent with the exploratory nature of subgroup identification in this sample. Post hoc pairwise comparisons with Bonferroni correction did not identify any community pairs with significantly different clinical profiles, suggesting that the observed numerical differences should be interpreted cautiously and require replication in larger samples.

Community profiles revealed notable patterns beyond the diagnostic composition (Table 3). **Community 1** comprised the oldest participants (approximately 11–16 years older than the other communities) with moderate symptom severity and the highest cognitive scores, slightly above the population mean. **Community 2**, the smallest community, showed the most severe symptoms (moderate-to-severe range) and was notably the youngest, with below-average cognitive scores. **Community 3** had the most balanced IBS/HC composition and the lowest symptom severity (mild range), with cognitive performance close to the population mean. **Community 4** exhibited the lowest cognitive scores (approximately one standard deviation below the population mean), suggesting a subgroup characterized by cognitive impairment alongside high IBS prevalence and moderate-to-severe symptoms. These patterns suggest that communities are differentiated not only by brain–cognition similarity but also by clinically meaningful characteristics including age, symptom burden, and cognitive function.

To further characterize the brain–cognition profiles distinguishing each community, we computed mean z-scores for all 42 features within each community (Table 4). **Community 1** (oldest, preserved cognition) showed the largest estimated intracranial volume and elevated cognitive scores, but markedly reduced gray matter volumes, suggesting preserved cognitive function despite smaller brain tissue volumes relative to intracranial capacity. **Community 2** (youngest, most severe IBS symptoms) exhibited elevated bilateral cortical volumes but substantially reduced white matter and corpus callosum volumes, potentially reflecting a developmental pattern with robust cortical gray matter but less white matter integrity. Notably, Community 2’s most distinguishing features were exclusively morphometric, suggesting that this young, high-symptom subgroup is characterized primarily by brain structural patterns rather than cognitive differences. **Community 3** (most balanced IBS/HC ratio, mildest symptoms) showed the largest subcortical gray matter volumes with particularly elevated basal ganglia structures, alongside the smallest intracranial volumes. **Community 4** (lowest cognitive scores) displayed reduced performance across multiple cognitive domains alongside elevated corpus callosum and putamen volumes, demonstrating that larger white matter structures do not necessarily correspond to better cognitive performance. These feature profiles suggest that communities capture distinct neurobiological patterns characterized by different patterns of brain structure–cognition relationships, which may have implications for understanding IBS heterogeneity and potentially for tailoring interventions. These communities should be interpreted as hypothesis-generating patterns rather than established subtypes, requiring validation in independent cohorts before clinical application.

### 3.7. Network Robustness to Edge Removal

**Figure 3:**
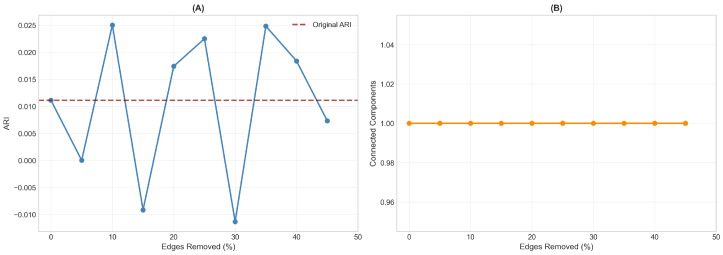
Network robustness to edge removal. (**A**) Adjusted Rand Index (ARI) values as edges are progressively removed from 0% to 45% (solid blue line). The dashed red line indicates the original ARI (0.011). The high variability ranging from approximately −0.010 to +0.025 with no systematic trend suggests that the modest community-diagnosis correspondence reflects chance variation rather than true structure. (**B**) Number of connected components in the network under edge removal. The network maintains a single connected component throughout, demonstrating that the underlying network topology based on brain–cognition similarity is robust even when community assignments are unstable. This dissociation indicates that patients are reliably connected based on their multivariate profiles, but these connections do not partition into communities that align with diagnostic categories.

Network robustness was assessed by progressively removing up to 45% of edges randomly and re-running community detection (Figure 3). The ARI values fluctuated considerably under perturbation, ranging from approximately −0.010 to +0.025, with no systematic trend relative to the original ARI of 0.011. This instability indicates that the modest community-diagnosis correspondence observed in the primary analysis likely reflects chance variation rather than true structure. In contrast, network connectivity remained stable throughout, with the network maintaining a single connected component even at 45% edge removal. This dissociation (unstable community assignments but robust network topology) suggests that while patients are reliably connected based on their brain–cognition profiles, the grouping of these connections into communities does not capture a meaningful diagnostic distinction.

### 3.8. Graphical Summary

**Figure 4:**
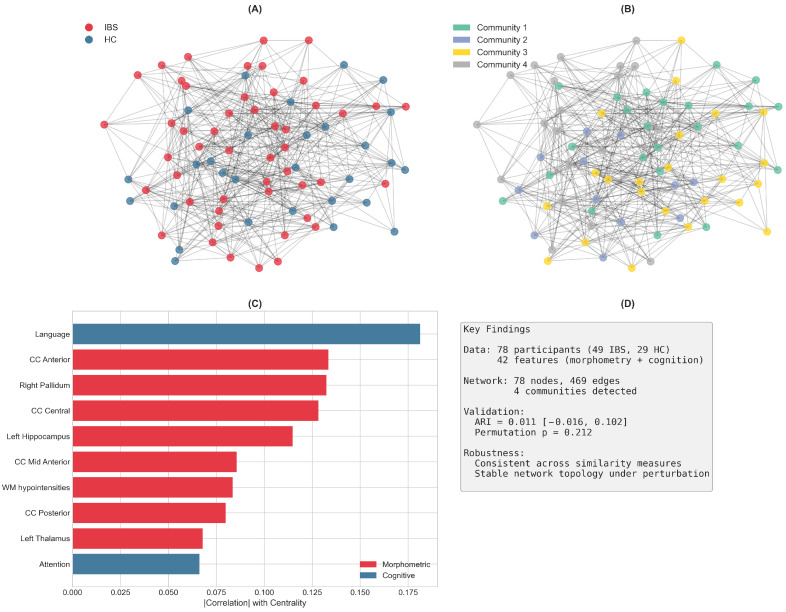
Graphical summary of the Patient Similarity Network analysis. (**A**) The PSN with nodes colored by diagnostic group (IBS = red, HC = blue), showing the mixing of patients across the network. (**B**) The same network with nodes colored by the four detected communities, illustrating how the Louvain algorithm partitions patients based on brain–cognition similarity rather than diagnosis. (**C**) Top 10 features contributing to network structure, with morphometric features (red, n = 8) and cognitive features (blue, n = 2) both represented corpus callosum segments, subcortical structures (pallidum, thalamus, hippocampus), and cognitive indices (Language, Visuospatial). (**D**) Key quantitative findings: the network comprises 78 nodes and 469 edges; community detection yielded an ARI of 0.011 with 95% confidence interval spanning zero; permutation testing confirmed non-significant correspondence with diagnosis (*p* = 0.212); and results were consistent across similarity measures and robust to network perturbation. Each detected community exhibited distinct brain–cognition feature profiles (Table 4), with differences in corpus callosum volumes, subcortical structures, and cognitive indices (particularly Visuospatial and memory domains) characterizing the communities. **Key Findings:** (1) PSN reveals 4 communities with distinct brain–cognition profiles; (2) Communities do not align with IBS/HC diagnosis (ARI ≈ 0, *p* = 0.21); (3) Each community shows unique patterns of brain structure and cognitive function; (4) Results robust across similarity measures and network perturbation; (5) Findings suggest brain–cognition heterogeneity cuts across diagnostic categories.

Figure 4 provides a graphical overview of the main findings from this PSN analysis.

## 4. Discussion

Irritable Bowel Syndrome presents a significant challenge for precision medicine due to its heterogeneous clinical presentation and the complex interplay between gastrointestinal symptoms, brain structure, and cognitive function. Traditional diagnostic approaches classify patients based on symptom criteria (Rome IV) and severity scores (IBS-SSS), yet these categories may not capture the underlying neurobiological diversity that could inform personalized treatment strategies. This study was motivated by the need for data-driven approaches that can reveal natural patient groupings based on objective biological and cognitive measures, independent of symptom-based classification.

We employed Patient Similarity Networks to investigate whether brain morphometry and cognitive features partition patients in ways that align with, or transcend, clinical diagnostic boundaries. This unsupervised approach allows patient subgroups to emerge from similarity structure rather than predefined categories.

The present study uses exactly the same dataset and feature set as Lundervold et al. [33], enabling direct methodological comparison. That study applied supervised machine learning (XGBoost, Random Forest) to classify IBS patients versus healthy controls, achieving 93% sensitivity by optimizing decision boundaries that maximally separate the two groups. In contrast, our PSN approach asks a fundamentally different question: rather than “can we distinguish IBS from HC?”, we ask “how do patients naturally cluster based on their brain–cognition profiles, and do these clusters correspond to diagnostic categories?” Supervised ML learns discriminative features that differ between groups; PSN reveals similarity structure across the entire cohort without regard to labels. These complementary perspectives (one optimized for classification, the other for structure discovery) together provide a more complete picture of patient heterogeneity than either approach alone.

Our analytical pipeline proceeded in four stages: (1) network construction from 42 features (36 morphometric, 6 cognitive) using Gaussian kernel-transformed Euclidean distances; (2) community detection via Louvain modularity optimization to identify patient subgroups; (3) centrality analysis to identify influential patients and features; (4) independent validation against clinical measures (IBS-SSS) that were deliberately excluded from network construction to avoid circularity.

### 4.1. Main Findings

The PSN approach yielded four communities that did not correspond to diagnostic groups (ARI near zero, permutation p=0.212), yet each exhibited distinct brain–cognition profiles (Table 4). Communities differed systematically in age, symptom severity, cognitive performance, and brain structural patterns. The relationship between brain structure and cognition varied across communities: some showed preserved cognition despite reduced brain volumes, while others displayed the opposite pattern. Notably, Community 2 (youngest, most severe symptoms) was distinguished exclusively by morphometric features, suggesting that symptom severity in this subgroup may be driven by brain structural differences rather than cognitive impairment.

Critically, the PSN captures a brain–cognitive dimension that is independent of symptom severity: because IBS-SSS was excluded from network construction, the detected communities reflect patient similarity based purely on objective neurobiological and cognitive features. This design allows us to assess whether brain–cognition patterns align with, or cut across, symptom-based classification, and our results demonstrate the latter.

This result is consistent with the heterogeneous nature of IBS as a clinical syndrome. The disorder encompasses patients with diverse symptom profiles, varying degrees of cognitive involvement [9,10], and likely different underlying pathophysiological mechanisms [17,40]. A network constructed from morphometric and cognitive features would be expected to reflect this heterogeneity rather than reproduce a binary diagnostic distinction. Indeed, contemporary conceptualizations of IBS emphasize that it represents a spectrum of disorders unified by symptom-based criteria rather than a single pathophysiological entity [41,42].

### 4.2. Relation to Prior Research

Our findings complement and extend the machine learning approaches developed within the B-BGM project. Lundervold et al. [33] achieved 93% sensitivity for IBS classification using XGBoost and Random Forest classifiers with the same feature set, while a companion study [16] successfully decoded psychological distress using similar machine learning methodology (see also [43] regarding assessment of self-reported executive function in IBS using a machine learning framework). The apparent discrepancy between high classification accuracy and low community-diagnosis correspondence reflects the fundamental difference between supervised and unsupervised approaches. Supervised classifiers are optimized to find discriminative boundaries between groups, whereas unsupervised community detection identifies natural clusters based on overall similarity patterns without regard to group labels. This distinction has been noted in other applications of PSN to clinical populations [21,22].

The convergent evidence regarding feature importance between the machine learning analysis [33] and our PSN centrality correlations is noteworthy. Both approaches highlight subcortical structures, particularly the hippocampus, as influential features. This aligns with a growing body of neuroimaging research demonstrating structural and functional alterations in limbic regions among IBS patients [12,44,45]. The hippocampus, in particular, has been implicated in stress-related modulation of visceral pain processing and emotional regulation in IBS [46,47]. Similarly, cognitive domains emerged as important in both analyses, consistent with reports of cognitive involvement in disorders of gut–brain interaction [8]. Notably, Billing et al. [10] found that cognitive impairment in IBS is only weakly correlated with anxiety/depression and GI symptoms, suggesting that cognition represents an independent dimension of the disorder that may contribute to the subgroup structure observed in our network analysis.

The brain–gut axis framework provides important context for interpreting these findings. Mayer et al. [6] articulated a systems view of IBS emphasizing that the disorder arises from dysregulated brain–gut–microbiome interactions, advocating for analytical approaches that capture this complexity. Bidirectional communication between the central nervous system and the gastrointestinal tract involves neural, hormonal, and immune pathways [5,48]. Neuroimaging studies have consistently demonstrated altered brain structure and function in IBS, particularly in regions involved in visceral sensation, emotional processing, and cognitive control [11,13,49]. The 12-year prospective study by Koloski et al. [50] provided compelling evidence for bidirectional brain–gut pathways, showing that psychological distress predicts subsequent GI symptoms and vice versa. Our PSN results, by identifying communities that blend IBS and HC participants based on brain–cognition profiles, may reflect these shared neural substrates: rather than forming distinct clusters, IBS patients and healthy controls exhibit overlapping brain–cognition patterns.

Together, the supervised classification results from Lundervold et al. [33] and our unsupervised PSN analysis provide a more complete picture: high classification accuracy demonstrates that IBS patients do differ from controls on average, while the mixing of groups within PSN communities reveals substantial overlap in brain–cognition profiles at the individual level.

### 4.3. Clinical Implications

Current treatment guidelines for IBS, including those from NICE [51] and the Rome Foundation, recommend a stepwise approach applicable to all patients with DGBI. This approach progresses from patient education and dietary adjustments, through structured interventions such as the FODMAP diet and symptom-directed pharmacotherapy, to central neuromodulators [52] and psychological interventions for severe or refractory cases. The Rome Foundation’s Multidimensional Clinical Profile [18] provides a framework for individualizing treatment based on diagnosis, symptom subtype, quality of life impact, psychological comorbidities, and physiological disturbances [17].

The present findings suggest that this severity-based model does not capture the heterogeneity within IBS populations. All patients in this study had moderate-to-severe IBS at baseline, meaning that even Community 3, characterized by the “mildest” symptoms, still represents at least a moderate symptom burden. Yet, our PSN analysis revealed four communities with markedly different brain–cognition profiles. Notably, Community 2 and Community 4 both showed high IBS prevalence and substantial symptom severity, but exhibited distinct neurobiological signatures where Community 2 was characterized by reduced white matter with preserved cognition, and Community 4 displayed cognitive impairment across multiple domains. These findings demonstrate that “severe IBS” is not homogeneous from a neurobiological perspective.

Cognitive function is not explicitly included in the MDCP framework, despite evidence that cognitive impairment is common in IBS and only weakly correlated with anxiety, depression, and GI symptom severity [10]. The identification of a subgroup with pronounced cognitive difficulties raises the hypothesis that such patients might benefit from earlier introduction of interventions with demonstrated cognitive effects in IBS, such as cognitive behavioral therapy or gut-directed hypnotherapy [42,53], rather than reserving these for refractory cases.

Several limitations preclude clinical application. The modest community sizes, non-significant statistical tests after correction, and instability of community assignments under perturbation indicate that these patterns require replication. Moreover, the cross-sectional design cannot establish the nature of the observed differences. The distinct profiles could represent pre-existing traits conferring vulnerability to IBS, acquired consequences of living with chronic DGBI, or downstream effects of shared underlying factors such as genetics, early life adversity, or microbiome composition. Distinguishing between these possibilities requires longitudinal studies.

These findings suggest that neurobiological subtyping based on brain morphometry and cognition could meaningfully complement symptom-based severity grading in IBS, revealing heterogeneity that current frameworks obscure. However, before clinical translation, prospective studies are needed to determine whether these neurobiologically-defined subgroups respond differently to targeted interventions.

### 4.4. Strengths and Limitations

This study has several notable methodological strengths that merit consideration in the context of the broader PSN literature. The use of multiple similarity measures (Euclidean, cosine, and correlation distances) demonstrated robustness of the network structure to methodological choices, addressing the known issue that network construction results can vary substantially depending on the similarity metric used [19]. Unlike many PSN applications that rely on a single distance metric, our multi-metric approach provides stronger evidence that observed patterns are not artifacts of the similarity measure chosen.

Rigorous statistical validation through bootstrap confidence intervals and permutation testing provided appropriate uncertainty quantification, a feature often lacking in PSN studies. The bootstrap approach accounts for sampling variability, while permutation testing addresses the multiple comparisons inherent in community detection [20]. The comparison with established machine learning methods from the same dataset enabled meaningful methodological synthesis, demonstrating both convergent and complementary insights from supervised and unsupervised approaches.

The combination of brain morphometry with cognitive measures leverages complementary information sources relevant to the gut–brain axis, extending beyond purely imaging-based or symptom-based approaches. This multi-modal feature set, while limited compared to studies incorporating genetics, microbiome, or metabolomics [13,54], provides a clinically accessible foundation for patient characterization. The current PSN was constructed exclusively from brain morphometry and cognitive measures. Other domains relevant to the gut–brain axis (including gut microbiome composition, which was collected as part of the B-BGM protocol, as well as inflammatory markers and autonomic function) were not incorporated. While this focused approach enables clear interpretation of brain–cognition similarity patterns, future studies integrating these additional modalities through approaches such as Similarity Network Fusion could provide a more comprehensive characterization of IBS heterogeneity.

Several limitations should be acknowledged. The sample size of 78 participants is comparable to many neuroimaging studies of IBS [46,47,55], but limits statistical power for subgroup analyses and may not fully capture the heterogeneity present in the broader IBS population. This power limitation was evident in our statistical testing: while trends in clinical variables (IBS-SSS, RBANS) across communities were observed, formal statistical tests (Kruskal–Wallis, chi-square) did not reach significance, and no features survived FDR correction for multiple testing. PSN studies in oncology and diabetes have typically employed larger cohorts (hundreds to thousands of patients) [21,22], enabling finer-grained subtyping that may require similar sample sizes in future IBS applications.

The cross-sectional design precludes assessment of temporal dynamics or trajectory-based patient groupings. Longitudinal studies have proven valuable in IBS research [50,55], and temporal PSN approaches could reveal dynamic patterns of patient similarity over time. As a single-site study using data from the B-BGM project, replication in independent cohorts from different populations and healthcare settings would strengthen generalizability. Importantly, the PSN-derived communities have not been externally validated in independent cohorts. Replication in separate IBS populations from different geographic and clinical settings is essential before these findings can inform clinical practice, consistent with best practices in network-based and machine learning studies.

Community detection methods involve inherent parameter choices (algorithm, resolution parameter) that can influence results. While the Louvain algorithm is widely used and computationally efficient [38], alternative methods such as spectral clustering or stochastic block models might yield different partitions. Our sensitivity analyses across parameter settings suggest reasonable stability, but the identified communities should be interpreted as one plausible partitioning among several consistent with the data.

### 4.5. Future Perspectives

Several promising directions merit future investigation to extend the PSN approach for IBS and related gut–brain disorders.

**Longitudinal and dynamic networks.** Tracking patient trajectories over time through longitudinal PSN analysis could identify patients who transition between communities, characterize stable versus dynamic phenotypes, and relate network position changes to clinical outcomes. Such approaches have proven valuable in other chronic conditions [22] and would leverage the longitudinal design available in studies like the B-BGM project.

**Multi-modal data integration.** Integration with gut microbiome data, which was collected as part of the B-BGM project protocol [32], could enable multi-modal networks capturing the full spectrum of gut–brain interactions [13,54]. Similarity Network Fusion (SNF) and related methods [19] provide principled approaches for combining heterogeneous data types into unified patient networks. Including genetic, metabolomic, and dietary information would further enrich patient characterization and align with the multi-omics paradigm in precision medicine [23,25].

**Artificial intelligence and deep learning extensions.** The intersection of network science and artificial intelligence offers exciting opportunities for advancing PSN methodology. Graph neural networks (GNNs) can learn patient representations directly from the network structure, potentially capturing complex patterns missed by traditional community detection [56]. Deep learning approaches have shown promise in medical imaging and clinical prediction [27,30], and their integration with network-based patient modeling could enhance both representation learning and outcome prediction. Attention mechanisms could identify which patient–patient relationships are most informative for clinical outcomes, providing interpretable insights alongside predictive power.

**Clinical decision support and personalized medicine.** Development of PSN-based clinical decision support tools could translate network insights into practical applications for patient stratification and treatment selection. Network position metrics could serve as biomarkers for prognosis or treatment response, complementing symptom-based assessment [24]. The goal of matching individual patients to optimal treatments, a cornerstone of precision medicine [23,57], may be advanced by understanding how patients cluster in multivariate feature space rather than relying solely on categorical diagnoses.

**Network medicine and disease module identification.** Integration with protein–protein interaction networks and disease modules [28,58,59] could connect patient-level networks to molecular mechanisms. This multi-scale approach, linking molecular networks to patient networks, represents a frontier in network medicine with potential to bridge mechanistic understanding and clinical phenotyping.

**Validation and clinical translation.** Rigorous validation in larger, independent IBS cohorts remains essential before clinical translation. Prospective studies testing whether network-derived subgroups predict treatment response would provide the strongest evidence for clinical utility. International collaborations pooling data across centers would enhance sample sizes and address generalizability. Ultimately, demonstrating that PSN-based stratification improves patient outcomes compared to standard care will be necessary for adoption in clinical practice [31].

## 5. Conclusions

This study demonstrates the utility of Patient Similarity Networks for investigating heterogeneity in Irritable Bowel Syndrome based on brain morphometry and cognitive features. While detected communities did not significantly align with diagnostic categories, they revealed clinically relevant subgroups with distinct symptom severity and cognitive profiles. The consistency of results across similarity measures and the convergence with prior machine learning findings support the robustness and relevance of the network-based approach. The PSN methodology offers unique insights into patient relationships and heterogeneity that complement traditional classification approaches, providing a foundation for future work on patient stratification and personalized management of this complex, heterogeneous gut–brain disorder. Importantly, the PSN methodology captures brain–cognition similarity independent of symptom severity, offering a complementary perspective to symptom-based approaches.

## Figures and Tables

**Table 1:** Comparison of similarity measures for PSN construction.

Metric	Edges	Communities	ARI	NMI
Euclidean	469	4	0.011	0.037
Cosine	418	4	−0.017	0.007
Correlation	391	4	0.006	0.037

**Table 2:** Centrality measures by group (mean ± SD).

Group	Degree	Betweenness	Eigenvector
IBS	0.155±0.061	0.015±0.018	0.104±0.055
HC	0.159±0.058	0.018±0.015	0.096±0.045

**Table 3:** Clinical and demographic profiles of detected communities. Values are mean ± SD, reported on original measurement scales (not z-transformed). IBS-SSS = IBS Severity Scoring System (range 0–500); RBANS = Repeatable Battery for the Assessment of Neuropsychological Status Total Scale score (population mean = 100, SD = 15).

Community	N	IBS	HC	% IBS	Age	IBS-SSS	RBANS
1	23	15	8	65.2	43.7±10.1	179.3±128.4	101.8±13.4
2	12	9	3	75.0	27.7±8.3	240.6±140.6	92.0±15.8
3	23	10	13	43.5	32.5±11.9	134.1±135.1	99.5±9.8
4	20	15	5	75.0	33.8±8.3	208.8±123.8	84.3±10.9

**Table 4:** Distinguishing features of detected communities. Mean Z = average standardized score for that feature within the community, computed from z-score transformed data (mean = 0, SD = 1 across all participants). Positive values indicate the community average is elevated relative to the overall sample mean; negative values indicate it is reduced. M = Morphometric; C = Cognitive. For each community, features were selected by ranking all 42 features by mean z-score and displaying the three most elevated (highest positive z-scores) and three most reduced (most negative z-scores). Note that Community 2 contains only morphometric features, indicating that its distinguishing characteristics are primarily structural rather than cognitive.

Community	Feature	Type	Mean Z
1 (n = 23)	eTIV	M	+0.61
	Total Scale (RBANS)	C	+0.48
	Visuospatial Index	C	+0.41
	Subcortical Gray Vol	M	−1.14
	Total Gray Vol	M	−1.12
	Cortex Vol	M	−1.07
2 (n = 12)	Left Cortex Vol	M	+0.72
	Cortex Vol	M	+0.70
	Right Cortex Vol	M	+0.68
	Right Cerebral WM Vol	M	−0.89
	Cerebral WM Vol	M	−0.89
	CC Anterior	M	−0.78
3 (n = 23)	Subcortical Gray Vol	M	+1.08
	Left Pallidum	M	+0.98
	Right Pallidum	M	+0.93
	eTIV	M	−0.38
	Language Index	C	−0.12
	CSF	M	+0.07
4 (n = 20)	CC Mid-Anterior	M	+0.56
	CC Posterior	M	+0.53
	Right Putamen	M	+0.50
	Total Scale (RBANS)	C	−0.78
	Visuospatial Index	C	−0.62
	Immediate Memory Index	C	−0.53

## Data Availability

The complete analysis workflow is publicly available at https://github.com/arvidl/psn-ibs (accessed on 14 January 2026), comprising reproducible Jupyter notebooks containing all analysis code and visualizations, the cleaned dataset in CSV format, Conda environment configuration for exact replication, and source code for generating all tables and figures. The computational analyses were developed with assistance from Claude Opus 4.5 (Anthropic, San Francisco, CA, USA) integrated within Cursor (Anysphere Inc., San Francisco, CA, USA).

## Supplementary Materials

The following supporting information can be downloaded at https://www.mdpi.com/article/10.3390/diagnostics16020357/s1. Table S1: Complete feature importance rankings for all 42 features; Table S2: Sensitivity analysis results across parameter combinations (k∈{5,8,10,15}, threshold ∈{0.2,0.3,0.4,0.5}); Table S3: RBANS Total Scale sensitivity analysis comparing results with and without the composite cognitive score; Figure S1: Heatmap of community feature profiles showing mean z-scores for top 20 most variable features across communities.

## Abbreviations

The following abbreviations and terms are used in this manuscript:

Adjacency matrix	Matrix representation of network connections
ARI	Adjusted Rand Index, clustering agreement measure adjusted for chance
B-BGM	Bergen Brain–Gut Microbiota project
Betweenness centrality	Measure of how often a node lies on shortest paths between other nodes
Bootstrap	Resampling method for estimating confidence intervals
CBT	Cognitive Behavioral Therapy
CC	Corpus Callosum
CI	Confidence Interval
Community	Group of nodes more densely connected to
	each other than to the rest of the network
Degree centrality	Normalized measure of node connectivity
DGBI	Disorder of Gut–Brain Interaction
Edge	Connection between two nodes representing patient similarity
Eigenvector centrality	Node importance based on connections to other important nodes
eTIV	Estimated total intracranial volume
FDR	False Discovery Rate, correction for multiple testing
FODMAP	Fermentable Oligosaccharides, Disaccharides,
	Monosaccharides, and Polyols
FreeSurfer	Neuroimaging software for brain segmentation and volumetric analysis
Gaussian kernel	Function transforming distances to similarities
GI	Gastrointestinal
Graph/Network	Mathematical structure of nodes connected by edges
HC	Healthy Controls
IBS	Irritable Bowel Syndrome
IBS-C/-D/-M	IBS subtypes: constipation-predominant, diarrhea-predominant, mixed
IBS-SSS	IBS Severity Scoring System (range 0–500)
k-NN	k-nearest neighbors, network construction method
Louvain algorithm	Greedy algorithm for community detection via modularity maximization
MDCP	The Rome Foundation’s Multidimensional Clinical Profile
MRI	Magnetic Resonance Imaging
Modularity (Q)	Measure of network partition quality
Network density	Proportion of possible edges present in the network
Network sparsification	Process of removing weak edges
NICE	National Institute for Health and Care Excellence
NMI	Normalized Mutual Information, clustering similarity measure
Node	Vertex in the network representing an individual patient
Node degree	Number of edges connected to a node
Permutation test	Non-parametric method using random label shuffling
PSN	Patient Similarity Network
RBANS	Repeatable Battery for the Assessment of Neuropsychological Status
SD	Standard Deviation
Similarity matrix	Matrix containing pairwise similarity values between patients
Similarity threshold	Minimum similarity for edge inclusion
SNF	Similarity Network Fusion
Weighted edge	Edge with numerical value representing similarity strength
