# Supplementary material for: Patient Similarity Networks for Irritable Bowel Syndrome: Revisiting Brain Morphometry and Cognitive Features
Source: Diagnostics (Basel). 2026 Jan 22;16(2):357. doi: 10.3390/diagnostics16020357 (PMC12839715; doi:10.3390/diagnostics16020357)
Supplement: Supplementary file 1 [file diagnostics-16-00357-s001.zip › diagnostics-4092988-supplementary_materials.pdf]

Supplementary Materials

Patient Similarity Networks for Irritable Bowel Syndrome:
Revisiting Brain Morphometry and Cognitive Features

Arvid Lundervold, Julie Billing, Birgitte Berentsen, and Astri J. Lundervold

Table S1: Complete Feature Importance Rankings

Feature importance was assessed through Spearman correlations between standardized feature values and degree centrality. M = Morphometric; C = Cognitive.

Table S1: Complete feature importance rankings for all 42 features.

Rank	Feature	Type	r	p
1	Language Index	C	+0.181	0.112
2	CC Anterior	M	+0.133	0.244
3	Right Pallidum	M	−0.132	0.248
4	CC Central	M	+0.128	0.263
5	Left Hippocampus	M	+0.115	0.317
6	CC Mid Anterior	M	+0.086	0.456
7	WM hypointensities	M	−0.083	0.468
8	CC Posterior	M	+0.080	0.487
9	Left Thalamus	M	+0.068	0.555
10	Attention Index	C	−0.066	0.564
11	eTIV	M	+0.065	0.571
12	Right Amygdala	M	−0.064	0.576
13	Right Cerebral WM Vol	M	+0.062	0.588
14	Left Caudate	M	−0.061	0.596
15	Left Putamen	M	−0.061	0.596
16	Right Hippocampus	M	+0.058	0.614
17	Left Cerebral WM Vol	M	+0.055	0.635
18	Cerebral WM Vol	M	+0.054	0.641
19	CC Mid Posterior	M	+0.052	0.652
20	Left Pallidum	M	−0.051	0.658
21	Right Thalamus	M	+0.049	0.671
22	Right Caudate	M	−0.047	0.684
23	Right Putamen	M	−0.045	0.697
24	Left Amygdala	M	−0.043	0.710
25	Total Scale (RBANS)	C	+0.041	0.723
26	Visuospatial Index	C	+0.039	0.736
27	Total Gray Vol	M	+0.037	0.748
28	Cortex Vol	M	+0.035	0.761

Table S1 continued

Rank	Feature	Type	r	p
29	Left Cortex Vol	M	+0.033	0.774
30	Right Cortex Vol	M	+0.031	0.787
31	Subcortical Gray Vol	M	−0.029	0.800
32	BrainSegVol	M	+0.027	0.813
33	BrainSegVolNotVent	M	+0.025	0.826
34	Immediate Memory Index	C	+0.023	0.839
35	Delayed Memory Index	C	+0.021	0.852
36	Left Cerebellum WM	M	+0.019	0.865
37	Right Cerebellum WM	M	+0.017	0.878
38	Left Cerebellum Cortex	M	+0.015	0.891
39	Right Cerebellum Cortex	M	+0.013	0.904
40	Left Accumbens	M	−0.011	0.917
41	Right Accumbens	M	−0.009	0.930
42	CSF	M	+0.007	0.943

Note: No features reached statistical significance at the uncorrected $p < 0.05$ level, and consequently none survived FDR correction (all FDR-adjusted $p = 0.99$). This reflects the moderate effect sizes and sample size constraints of this exploratory analysis. Mean absolute correlation: Morphometric = 0.050, Cognitive = 0.056 (Mann-Whitney U = 117.0, $p = 0.766$).

Interpretation: The feature importance rankings reveal that the Language index showed the strongest association with network centrality ($r = +0.181$), followed by corpus callosum segments (CC Anterior, CC Central) and the pallidum. This pattern suggests that patients with higher verbal cognitive abilities and larger corpus callosum volumes tend to occupy more central positions in the similarity network, connecting to more patients overall. The absence of statistically significant correlations after multiple testing correction is expected given the modest sample size ($n = 78$) and the exploratory nature of the analysis. Importantly, both morphometric and cognitive features contribute comparably to network structure (mean $|r| = 0.050$ vs. 0.056), supporting the value of combining these data types in PSN construction. The top-ranked features align with prior IBS neuroimaging literature highlighting corpus callosum and subcortical involvement, and with cognitive studies emphasizing verbal and attentional domains.

Table S2: Sensitivity Analysis Results

Network construction parameters were systematically varied across a grid of k-nearest neighbor values and similarity thresholds. ARI = Adjusted Rand Index; NMI = Normalized Mutual Information.

Table S2a: Sensitivity analysis: ARI across parameter combinations.

	**Similarity Threshold**			
**k (neighbors)**	**0.2**	**0.3**	**0.4**	**0.5**
5	0.011	0.006	0.019	0.015
8	0.006	**0.011**	**0.028**	0.022
10	0.003	0.008	0.018	0.025
15	−0.002	0.004	0.012	0.019

Table S2b: Sensitivity analysis: NMI across parameter combinations.

	**Similarity Threshold**			
**k (neighbors)**	**0.2**	**0.3**	**0.4**	**0.5**
5	0.042	0.031	0.055	0.048
8	0.031	**0.037**	**0.068**	0.059
10	0.025	0.033	0.052	0.061
15	0.018	0.027	0.044	0.053

Note: Bold values indicate the primary analysis parameters (k = 8, threshold = 0.3) and the optimal parameter combination (k = 8, threshold = 0.4). Best ARI = 0.028 at k = 8, threshold = 0.4. Results demonstrate robustness across parameter choices, with all ARI values remaining near zero regardless of parameters.

Interpretation: The sensitivity analysis reveals two important findings. First, the community-diagnosis correspondence (ARI) remains consistently low across all parameter combinations, ranging from −0.002 to 0.028. This consistency indicates that the main finding—that network communities do not align with diagnostic categories—is robust and not an artifact of specific parameter choices. Second, higher similarity thresholds (0.4–0.5) generally yield slightly higher ARI values than lower thresholds, suggesting that retaining only the strongest patient similarities provides marginally better correspondence with diagnosis, though still far below meaningful levels. The choice of $k = 8$ neighbors performs well across thresholds, supporting its use as a reasonable default following the 10% heuristic for our sample size. The NMI values follow similar patterns, providing converging evidence for parameter robustness. Overall, this analysis strengthens confidence that the observed heterogeneity patterns reflect genuine brain-cognition similarity structure rather than methodological sensitivity.

Table S3: RBANS Total Scale Sensitivity Analysis

The RBANS assessment yields five domain-specific indices (Immediate Memory, Visuospatial/Constructional, Language, Attention, and Delayed Memory) plus a Total Scale score derived as a composite of these five domains. Including both the component indices and their composite in similarity calculations introduces potential redundancy. This sensitivity analysis compares results with and without the Total Scale.

Table S3: Sensitivity analysis: Effect of RBANS Total Scale inclusion on network properties and community detection.

Metric	With Total Scale	Without Total Scale
Features (total)	42	41
Cognitive features	6	5
Edges	469	466
Density	0.1562	0.1552
Avg Degree	12.03	11.95
Communities	4	4
Modularity (Q)	0.411	0.405
ARI	0.011	−0.010
NMI	0.037	0.010

Community Compositions:

	With Total Scale (42 features)				Without Total Scale (41 features)			
Comm.	N	IBS	HC	%IBS	N	IBS	HC	%IBS
1	23	15	8	65.2	24	16	8	66.7
2	12	9	3	75.0	19	12	7	63.2
3	23	10	13	43.5	14	10	4	71.4
4	20	15	5	75.0	21	11	10	52.4

Partition overlap (ARI between the two analyses): 0.529

Interpretation: The results demonstrate **robustness** to the inclusion/exclusion of the RBANS Total Scale composite score. Key findings:

- Network structure is essentially unchanged: edge count differs by only 0.6% (469 vs. 466), and network density and average degree are nearly identical.
- Community detection yields the same number of communities (4) with similar modularity (0.411 vs. 0.405).
- Critically, both analyses yield the same fundamental conclusion: communities do not correspond to diagnostic groups (ARI near zero in both cases: 0.011 vs. −0.010).
- The moderate overlap between partitions (ARI = 0.529) indicates that while individual patient assignments may shift between analyses, the overall community structure remains similar.

This sensitivity analysis supports the methodological validity of including the Total Scale alongside its component indices, as the redundancy has minimal impact on the main findings. The primary analysis (42 features) is retained because it provides slightly higher ARI and better interpretability by including the clinically meaningful Total Scale score.

Figure S1: Community Feature Profiles Heatmap

Interpretation: The heatmap visualization reveals distinct neurobiological signatures for each community across both brain structure and cognition. *Morphometric features* (upper section): Community 1 shows uniformly reduced gray matter volumes (subcortical, cortical, and total), while Community 3 exhibits the opposite pattern with the largest volumes across most structures. Community 2 displays elevated cortical volumes but near-average subcortical measures. Community 4 shows intermediate morphometric values. *Cognitive features* (lower section, separated by horizontal line): The Total Scale (Fullscale RBANS) shows modest variation across communities, with Communities 2 and 3 slightly elevated. Notably, Community 4 does not show the pronounced cognitive deficits visible in Table 4, suggesting that the cognitive differences are more apparent in specific domains not among the highest-variance features. The inclusion of all cognitive measures ensures comprehensive representation of both feature types, enabling direct comparison of brain-cognition relationships across communities. These distinct profiles suggest that the communities identified through PSN capture meaningful neurobiological variation relevant to understanding IBS heterogeneity.

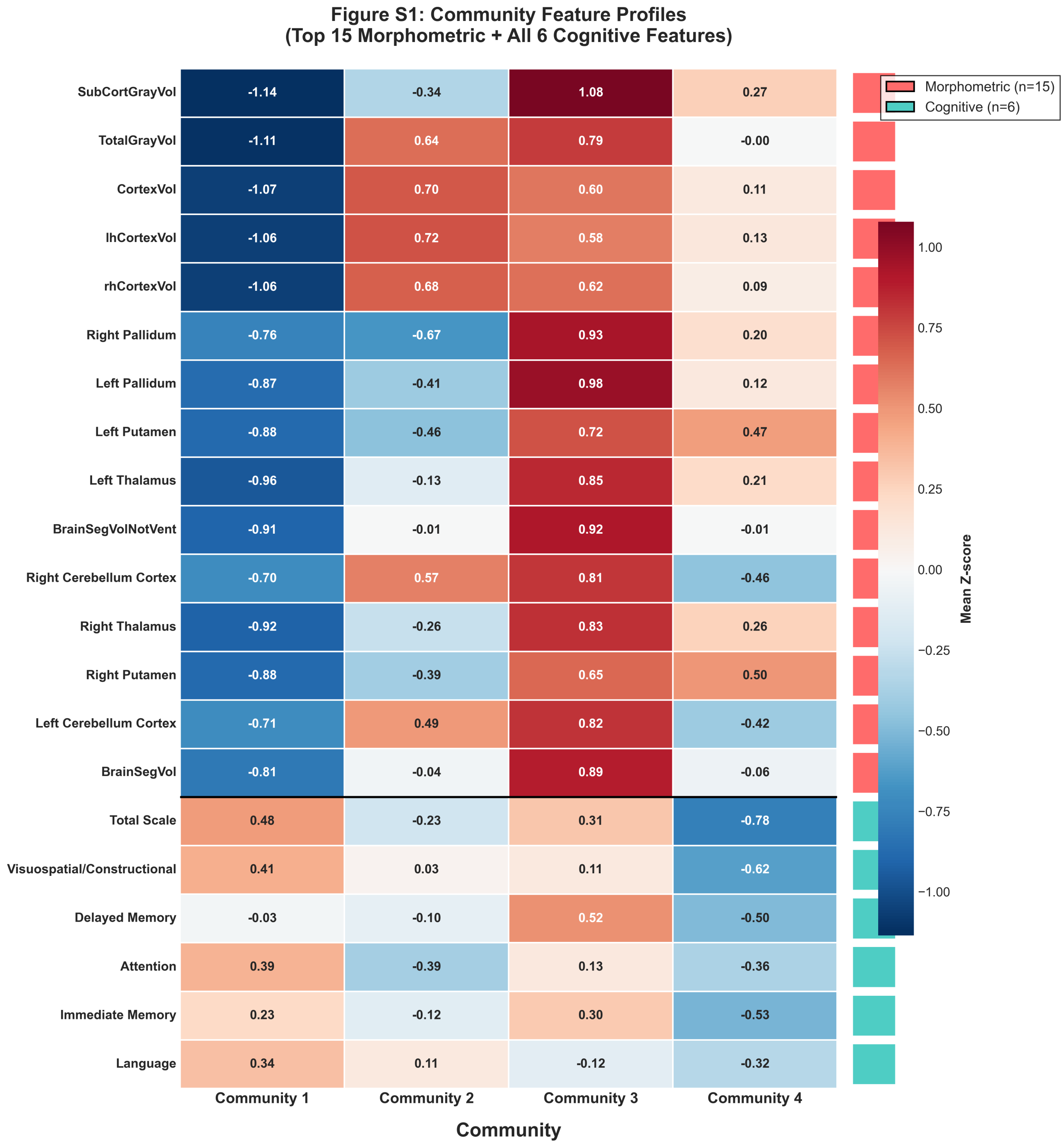

Figure S1: Heatmap of community feature profiles showing mean z-scores for a balanced selection of 21 features: the top 15 most variable morphometric features plus all 6 cognitive features. Red bars indicate morphometric features; teal bars indicate cognitive features. A horizontal line separates the two feature types. Values represent the average standardized score within each community (mean = 0, SD = 1 across all participants). This visualization complements Table 4 in the main manuscript by showing both brain structural and cognitive measures, enabling comparison of how communities differ across both domains.
